# A Novel Concept of a Phased-Array HIFU Transducer Optimized for MR-Guided Hepatic Ablation: Embodiment and First *In-Vivo* Studies

**DOI:** 10.3389/fonc.2022.899440

**Published:** 2022-05-30

**Authors:** Orane Lorton, Pauline C. Guillemin, Yacine M’Rad, Andrea Peloso, Sana Boudabbous, Caecilia Charbonnier, Ryan Holman, Lindsey A. Crowe, Laura Gui, Pierre-Alexandre Poletti, Alexis Ricoeur, Sylvain Terraz, Rares Salomir

**Affiliations:** ^1^Image Guided Interventions Laboratory (GR-949), Faculty of Medicine, University of Geneva, Geneva, Switzerland; ^2^Visceral Surgery Division, University Hospitals of Geneva, Geneva, Switzerland; ^3^Radiology Division, University Hospitals of Geneva, Geneva, Switzerland; ^4^Medical Research Department, Artanim Foundation, Geneva, Switzerland

**Keywords:** hepatic ablation, thermotherapy, HIFU, phased-array, MR thermometry

## Abstract

**Purpose:**

High-intensity focused ultrasound (HIFU) is challenging in the liver due to the respiratory motion and risks of near-/far-field burns, particularly on the ribs. We implemented a novel design of a HIFU phased-array transducer, dedicated to transcostal hepatic thermo-ablation. Due to its large acoustic window and strong focusing, the transducer should perform safely for this application.

**Material and Methods:**

The new HIFU transducer is composed of 256 elements distributed on 5 concentric segments of a specific radius (either 100, 111, or 125 mm). It has been optimally shaped to fit the abdominal wall. The shape and size of the acoustic elements were optimized for the largest emitting surface and the lowest symmetry. Calibration tests have been conducted on tissue-mimicking gels under 3-T magnetic resonance (MR) guidance. *In-vivo* MR-guided HIFU treatment was conducted in two pigs, aiming to create thermal ablation deep in the liver without significant side effects. Imaging follow-up was performed at D0 and D7. Sacrifice and post-mortem macroscopic examination occurred at D7, with the ablated tissue being fixed for pathology.

**Results:**

The device showed −3-dB focusing capacities in a volume of 27 × 46 × 50 mm^3^ as compared with the numerical simulation volume of 18 × 48 × 60 mm^3^. The shape of the focal area was in millimeter-range agreement with the numerical simulations. No interference was detected between the HIFU sonication and the MR acquisition. *In vivo*, the temperature elevation in perivascular liver parenchyma reached 28°C above physiological temperature, within one breath-hold. The lesion was visible on Gd contrast-enhanced MRI sequences and post-mortem examination. The non-perfused volume was found in pig #1 and pig #2 of 8/11, 6/8, and 7/7 mm along the LR, AP, and HF directions, respectively. No rib burns or other near-field side effects were visually observed on post-mortem gross examination. High-resolution contrast-enhanced 3D MRI indicated a minor lesion on the sternum.

**Conclusion:**

The performance of this new HIFU transducer has been demonstrated *in vitro* and *in vivo*. The transducer meets the requirement to perform thermal lesions in deep tissues, without the need for rib-sparing means.

## Introduction

The incidence of hepatic cancer has been rising sharply over the past 20 years and it is the sixth most commonly occurring cancer in patients. More than 800k people are diagnosed with this cancer each year throughout the world, and in 2018, it was the second leading cause of cancer death globally (1). Surgery and liver transplantation remain the gold standard treatment for patients with liver tumors, although the tumors located close to the portal and hepatic veins are often difficult to treat, and these can sometimes be inoperable (2–4). In recent decades, thermal ablative techniques, such as radiofrequency (RF), microwave (MW), high-intensity focused ultrasound (HIFU), or cryotherapy, are being increasingly recognized as treatment options for primary cancers (5–8), and radiofrequency ablation is even considered as a good alternative for small hepatocellular carcinomas in patients ineligible for liver transplantation (9). Metastatic disease can be particularly problematic due to its high prevalence, reported to be between 18 and 40 times more common than primary liver tumors (10). This is particularly significant for metastatic colorectal cancer, with more than 80% surgical ineligibility (11). The European Society for Medical Oncology recommends the consideration of ablative therapies in metastatic colorectal cancer cases presenting with surgical ineligibility or oligometastatic disease (12).

HIFU is a non-invasive technique that uses an array of external transducers that focus the beam on a prescribed target and was first described by Lynn et al. (13). This technique has been successfully tested in clinical studies for a wide variety of tumor types, including brain (14), prostate (15), and breast cancer (16), or bone metastases (17–19).

Several studies reported successful HIFU ablation in patients with non-resectable hepatocellular carcinoma (HCC) under ultrasound guidance (20, 21). The studies demonstrated that HIFU ablation can improve survival outcomes. However, ultrasound imaging does not offer temperature monitoring in a clinical setting, suffers from interference during sonications, and does not allow assessment of treatment efficacy during the procedure.

Since the 1990s, HIFU therapy has been coupled to magnetic resonance imaging (MRgHIFU) for guiding thermal therapies due to its ability to spatially map the local temperature distribution (22). Moreover, near real-time MR thermometry based on the proton frequency resonance shift (PRFS) can be used to create an automatic feedback control of the ultrasound beam to improve the efficacy and safety of the HIFU treatment. However, HIFU ablation for abdominal organs like the liver is more challenging due to the respiratory motion and the high perfusion, but most of all because the acoustic access is partially obstructed by the thoracic cage. Indeed, the ribs block a part of the acoustic energy delivered by the HIFU beam that can affect the focusing, and the exposure of the ribs to acoustic energy can damage them with overheating (21, 23) due to the high value of the absorption coefficient of the bone (24).

To avoid the ribs from heating, several methods have been investigated. The main objective is to find the balance between delivering enough acoustic energy through the rib cage to achieve the ablation of the target tissues while avoiding secondary heating, especially in the bones. Zhu et al. proposed to perform a partial rib resection prior to HIFU ablation of hepatocellular carcinoma to offer patients an improved prospect of complete tumor ablation (25). A single element transducer was used in this study. However, this method reduces the non-invasiveness of the HIFU therapy and increases the risk of infection.

In the 1990s, several studies proposed phased-array applicators to sonicate between the ribs or bones (26, 27). In recent years, many phased-array transducers have been developed (28, 29) and some have been used for liver ablation (11, 30–33). Salomir et al. (34) used reflective strips inserted in the pre-focal beam pathway, external to the body, to block the energy targeted on the ribs. Quesson et al. proposed a method where the ribs are visualized from anatomical MR data and projected onto the transducer surface to identify which elements have to be deactivated. This method was validated *ex vivo* and *in vivo* in pig liver during breathing under real-time MR thermometry (35). Ramaekers et al. designed a Voronoi-tessellated transducer based on Fermat’s spiral, compared the device to currently available clinical transducers, and showed that this design reduces acoustic energy deposit on the ribs and the energy density in the pre-focal zone (36).

Carling et al. (37) studied the possibility to deliver HIFU lesions near the portal or hepatic vein and also the effect of the ablation on the portal or hepatic vein. It was concluded that ablation of liver tissue can be performed adjacent to the hepatic or portal vein while keeping the vessel walls intact. Also, some USgFUS studies have reported the ability of ablation in difficult locations, such as near large vessels and near surrounding organs (38, 39).

To manage the respiratory motion, several methods were used, which can be distinguished into two solutions: 1) motion suppression with apnea or high-frequency jet ventilation and 2) motion tracking (40). Auboiroux et al. (41) suggested a gated sonication by tracking the thoracic wall motion using a modified MR-conditional optical camera. A software detected the respiratory motion in real time and the acoustic power was emitted in the lowest part of the exhalation phase. The self-scanning method described by Lorton et al. (42) uses motion tracking by ultrasound and modulates the HIFU power in real time to compensate for the effect of the tissue motion and standardize the delivered power to the target.

Very limited reports are currently available on clinical studies using MRgHIFU on liver or pancreas targets. The chosen tumors were systematically located below the rib line and the acoustic window avoided bowel loops. The ExAblate 2000 system from InSightec was used off-label in every reported study to date.

Okada et al. (43) reported in 2006 a case of hepatocellular carcinoma treated with MR-guided focused ultrasound with respiratory gating on a conscious patient. Gedroyc (44) and then Fisher et al. (45) reported a few cases of MRgHIFU treatment in the low liver or the left lobe through the epigastrium, under general anesthesia and controlled respiration. Anzidei et al. reported in 2014 a series of seven hepatic and two pancreatic treatments (46, 47) in easily accessible portions of the organ, again under general anesthesia and in carefully selected patients to avoid near-field obstacles.

These few available reports demonstrated significant potential and technical success in all patients, with 20% to 100% of the tumor volume ablated and without significant complications. In terms of clinical results, the MRgFUS treatment was reasonably successful for local tumor control or pain management. Nevertheless, the geometrical limitations prevented reaching lesions behind the ribs or lungs. To date, neither the MRgHIFU application to the liver and pancreas has emerged as a therapeutic option for a clinically relevant population of patients, nor an FDA/CE-approved device has been made available for consumer use.

In this study, we designed and validated a novel concept of a HIFU transducer dedicated to liver thermo-ablation. The novelty of this transducer is the large bend radius tailored to fit the abdominal wall, while the natural focus locates 10 cm deep inside the tissues, yielding a low effective F-number not reachable with single shell emitters. Furthermore, the device distributes the acoustic energy over a large entry window. The first step of this study was to determine the performance of the transducer and ensure that the technical requirements conform to the expectations. The second step was to perform a liver ablation *in vivo* in two pigs, to validate the efficiency of the device and to evaluate the potential adverse effects.

## Material and Methods

### Transducer Design

**Figure 1:**
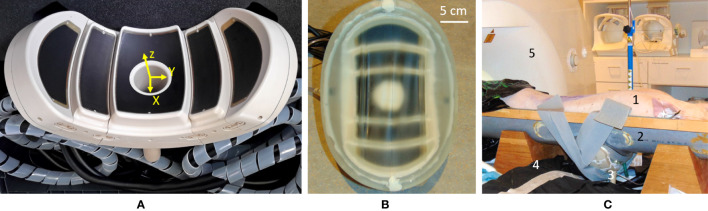
**(A)** Main view of the high-intensity focused ultrasound (HIFU) phased-array applicator. Note the five confocal spherical segments. **(B)** The HIFU transducer equipped with a semi-elastic membrane allowing the circulation of cooled water. **(C)** Overview of the *in-vivo* setup on the MR table: 1) prone-positioned animal; 2) semicylindrical support elevated to accommodate the transducer, under the animal; 3) the HIFU transducer with acoustic coupling; 4) one flexible MR surface coil; 5) 3-T MR scanner.

The transducer was designed by the authors and manufactured by the Imasonic company (Voray-sur-l’Ognon, France) to fit the abdominal morphology of the patients while concentrating the beam as deep as 10 cm. The working frequency is 650 kHz and the large acoustic window is achieved with 5 spherical segments of different radii, concentric to a unique natural focus (R1 = 100 mm, R2 = 111 mm, and R3 = 124 mm) (see **Figure 1A**). This configuration ensures an even energy distribution on a wide-body surface, aiming to avoid thermal buildup in the near field. The front side of the transducer was surrounded by a biocompatible membrane (**Figure 1B**) and filled with 3 L of cooled deionized circulating water at approximately 0.4 L/min to ensure ultrasound propagation and convective cooling of the transducer surface.

**Figure 2:**
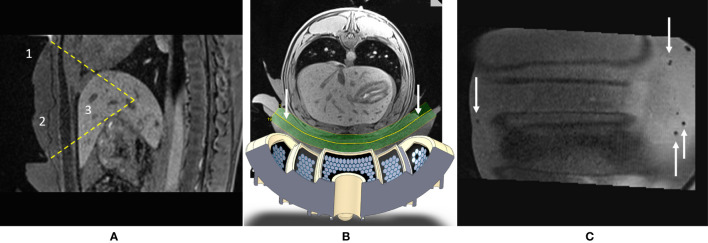
**(A)** Sagittal T1w MR image illustrating the targeting *in vivo.* Visible are the 1) HIFU transducer with its natural focal depth, 2) coupling layer, and 3) liver. Field of view (FOV) = 150 mm × 205 mm. **(B)** Augmented reality axial view of the setup: shown are the numerical design of the transducer and the geometry of the curved slice reconstruction of the acoustic window (see arrows). **(C)** Curved reconstruction image of the membrane-to-skin contact surface, demonstrating the absence of trapped air bubbles in the active aperture. Small bubbles can be seen outside the beam pathway. FOV = 175 mm × 188 mm.

The wide aperture due to the large bend radius enables abdominal fitting while keeping the natural focal point deep inside the tissues by natural geometric focusing (**Figure 2A**). Steering capabilities are further available for fine adjustment of the focus, motion tracking, and volumetric sonication. The preferential steering direction was chosen to be along the axial plane as the head–feet direction is easily manageable by manually sliding the transducer on the body. In this way, the elements were elongated along the *X*-axis of the transducer (**Figure 1A**) to maximize the number of elements in the *Y* direction and favor the steering along this axis. The active surface versus total surface ratio has been maximized to deliver the highest energy to the focal point by filling the surface with irregular hexagons of different sizes. The layout and size of the elements have been optimized to avoid symmetries and redundancies in order to minimize the secondary lobes and unexpected heating. The amplitude of RF excitation of each active element was weighted by the square root of that element surface, aiming for the generation of a uniform acoustic source. A cylindrical hole was installed at the center of the transducer to allow further use of ultrasound imaging as a means of quick targeting and motion tracking (48, 49). Ten thermocouples have been integrated into the piezocomposite material for temperature monitoring, ensuring safe use of the device, both for the material and the patient. Control measurements were performed using MR thermometry to confirm the precise mechanical alignment of the separate segments (concentric orientation and actual radii). The mechanical alignment was considered optimal if no further energy deposition at focus could be obtained by additional RF delays on the top of the electronic time-of-flight focusing applied to the transducer geometry.

### MRI Guidance

Experiments were performed under 3-T MR guidance (Prisma Fit, Siemens, Erlangen, Germany). The physical characterization of the transducer on phantoms relied on high-resolution 3D T1-weighted gradient recalled echo images for targeting (time of acquisition = 312 s, TR = 5.44 ms, TE = 1.91 ms, flip angle = 9.1°, BW = 390 Hz/pixel, acquisition resolution 0.9 × 0.8 × 1.34 mm^3^, reconstruction resolution 0.8 × 0.8 × 1.2 mm^3^) and PRFS MR thermometry for monitoring (three orthogonal slices acquired interleaved, segmented EPI factor = 7, temporal resolution = 2.7 s, FOV = 128 mm, slice thickness = 4 mm, in-plane resolution = 2 mm, TR = 50 ms, TE = 10 ms, flip angle = 20°, BW = 814 Hz/pixel, spectral-selective fat suppression). A combination of standard spine coil and flexible surface coil was used.

For the *in-vivo* experiments, pre-, per-, and postoperative images were acquired on a 3-T MR clinical scanner (Prisma Fit, Siemens, Erlangen, Germany), under forced apneas, by wrapping the animals in two flexible surface coils (see **Figure 1C**). Targeting and post-ablation imaging follow-up was performed using a breath-hold 3D T1-weighted gradient recalled echo sequence, with the following main acquisition parameters: time of acquisition = 54 s, TR = 4.08 ms, dual-echo Dixon water-fat separation TE1/TE2 = 1.27/2.5 ms, flip angle = 9.5°, BW = 1,040 Hz/pixel, parallel imaging GRAPPA factor = 2, reference lines = 24, acquisition resolution 1.6 × 1.2 × 1.74 mm^3^, and reconstruction resolution 1.2 × 1.2 × 1.4 mm^3^. Near real-time temperature monitoring was performed during breath-hold using the multislice PRFS sequence described above.

### Electronics

The described HIFU applicator was powered by a 256-channel beam former (Image Guided Therapy, Pessac, France), with the available frequency range of 500 kHz–3 MHz. Each channel is individually controllable and delivers up to 10 W electrical power on an adapted charge of 50 Ω.

### Software

The HIFU beam former was controlled by dedicated software *via* an ethernet connection. In-house software was written in Python under Windows 7 using the manufacturer libraries for driving the multichannel generator, as well as the Numpy library (https://numpy.org/). The software allowed the user to update the treatment parameters in real time such as the applied power, active channels, and eventual modulation of RF phases on the top of the focusing lookup table. The amplitude of each channel was automatically limited to a pre-defined threshold as a means of protection against transducer overheating. A built-in timer ensured that the sonication was active for a precise amount of time. Each sonication was monitored with embedded power meters channel-wise and the results were stored in log files.

### Physical Characterization of the Device

Transducer heating and quality control of focusing were assessed with the transducer immersed in a still cold-water bath. Performance and validation tests were performed on tissue-mimicking gels. The gel composition is as follows (42): 2% v/v agar, 8% v/v glycerol, and 12% v/v powder milk. Steering performance was assessed with MR thermometry. During the *in-vitro* experiments, the delivered acoustic energy ranged between 150 and 600 W and the duration of sonication ranged between 10 and 30 s.

The shape of the focal spot was determined using a thin hydrophone [sensor size 10 µm diameter (50)], maintained immobile at the focus in a dedicated 3D printed holder, during electronic steering over a few millimeters (acoustic scanning) of a low-power, short-pulse sonication. The scanning sonication (70 W during 1 ms) was steered in the three principal directions shown in **Figure 1A**.

Thermal map analysis was performed using Matlab R2016a (The MathWorks, Inc., Natick, MA, USA). The thermal effect was evaluated by calculating the relative temperature in the focal area, defined as a 4-pixel-diameter region of interest (ROI) around the warmest pixel. Data were corrected for the drift of the baseline temperature, defined as a mean temperature change in a larger ROI outside the HIFU beam pathway.

### *In-Vivo* Studies

*In-vivo* studies were performed on two female pigs, BW 26 and 37 kg, according to the ethical approval from the local animal research committee.

Premedication was performed 30 min before anesthesia by intramuscular injection of midazolam (0.75 mg/kg). A catheter was placed in an auricular vein and tracheal intubation was carried out. Ventilation was performed (Datex-Ohmeda F-CU8, Helsinki, Finland) at a frequency of 9–11 cycles per minute. Anesthesia was maintained by a 2% isoflurane (Abbott AG, Baar, Switzerland) air mixture and the following parameters were monitored: blood oxygenation rate, exhaled carbon dioxide, and heart rate. Every 30 min, the intra-auricular temperature was controlled with an MR-compatible thermometer. An external heating circuit was installed to help maintain the body temperature within physiological limits.

Between 3 and 5 min before the HIFU sonication, an intravenous injection of atracurium (0.6 mg/kg) was delivered in order to prevent any accidental muscle contraction during the HIFU ablation and to ensure a stable apnea. A Temgesic (Grunenthal, Aix-La-Chapelle, Germany) skin patch (0.01 mg/kg) is effective for 48 h and was applied prior to the animal’s wake-up.

The same general anesthesia protocol was performed 7 days after the operation for semichronic imaging control. An intravenous injection of gadolinium (0.3 ml/kg) was administered for contrast-enhanced MRI follow-up. The pig was sacrificed by an i.v. lethal dose of KCl. Necropsy and gross pathology were finally performed.

During the experiment, the animal lay in a prone position on a raised bed in the MR tunnel. The HIFU transducer was maintained under the pig in front of the liver (**Figure 1C**). This position ensured better stability of the MR thermometry and a larger acoustic window for sonicating the liver, as compared with the lateral decubitus or supine positions. The sonications were performed during breath-holds in the exhalation phase and ranged 30–40 s.

Acoustic coupling between the applicator’s membrane and the skin was achieved using standard ultrasonic gel and deionized degassed water. Air bubbles from the acoustic gel along the HIFU beam pathway were removed individually. Degassed water was added in small amounts on the top of the gel to smooth out any local irregularities on its surface, then the animal was laid down by two operators. The interface between the membrane and the skin was verified by reconstruction of a curved MR slice (**Figures 2B, C**).

The theoretical position of the focal point was determined using a geometrical model. Two MPR perpendicular planes (P1, P2) were positioned on the 3D T1w sequence, using the built-in tools of the MR scanner and corresponding to the mirror symmetry planes of the applicator. The intersection of P1 and P2 defined the main acoustic axis, where the natural focus should be found at a distance of 100 mm from the active surface. A third plane, P3, perpendicular both to P1 and P2, was defined at the level of the focal point. These three planes (P1, P2, P3) determined the orientation of the PRFS MR thermometry slices. If electronic steering of the beam was prescribed, the MR thermometry slices were shifted accordingly. The RF excitation of the three MR thermometry slices was performed interleaved; therefore, a partial saturation of the signal occurred at the crossing lines of these planes. While this effect is decreasing the local SNR, it is particularly useful to provide a landmark for the prescribed position of the focal point visible within the near real-time MR thermometry images and, subsequently, to enable its repositioning if mismatched.

Targeting was performed using a pilot sonication of 200 W during 30 s, and ablation was performed using 700–900 W during 30–40 s in a point-by-point serial sonication of 5 points cross pattern of 4 mm wide.

MRI follow-up was performed on days 0 and 7 based on the gadolinium contrast-enhanced 3D T1-weighted sequence described above.

Post-mortem, the abdomen was opened by a midline laparotomy followed by left and right lateral extension. A macroscopical inspection of the whole peritoneal cavity was performed. The falciform ligament was divided and the liver was then freed from the triangular ligaments. All peritoneal attachments were then divided and the liver was released from the diaphragm. The inferior cava and the infrahepatic cava were dissected and isolated. The liver was then freed by cutting all vascular attachments.

## Results

### Technical Performances

**Figure 3:**
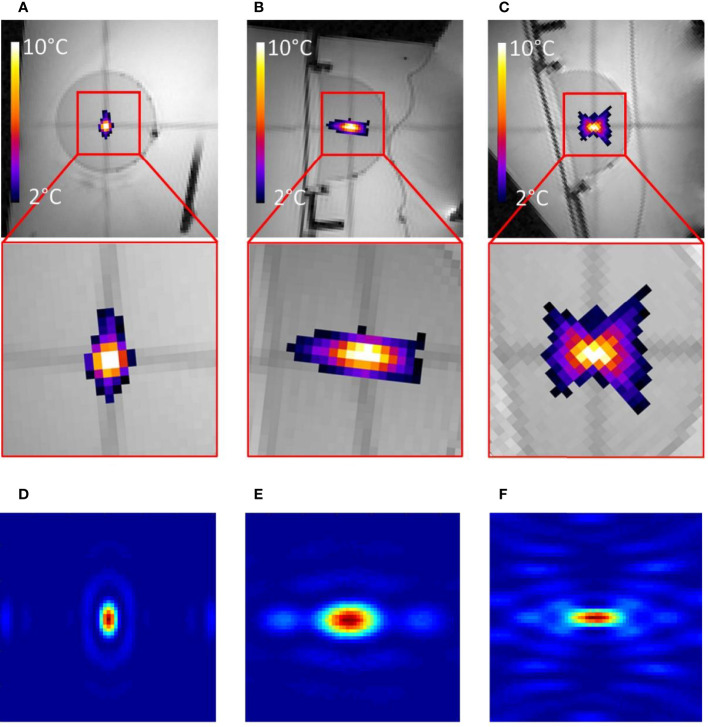
**(A–C)** MR images of the gel phantom merged with proton frequency resonance shift (PRFS) thermal maps in three orthogonal planes, during a 10-s HIFU sonication. The inset FOV is 55 mm and a color bar is provided for the temperature elevation. **(D–F)** Numerical calculation of acoustic intensity in three orthogonal planes; linear color scale; the FOV is 18 mm.

In terms of electronic beam forming, the novel HIFU transducer showed −3 dB steering capacities in a volume of 27 × 46 × 50 mm as compared with the numerical simulation volume of 18 × 48 × 60 mm. The temperature distribution around the focal area demonstrated a similar shape versus the acoustic intensity computation as illustrated in **Figure 3**. No electromagnetic interferences could be detected on MR data during concurrent HIFU sonication up to 900 W.

**Figure 4:**
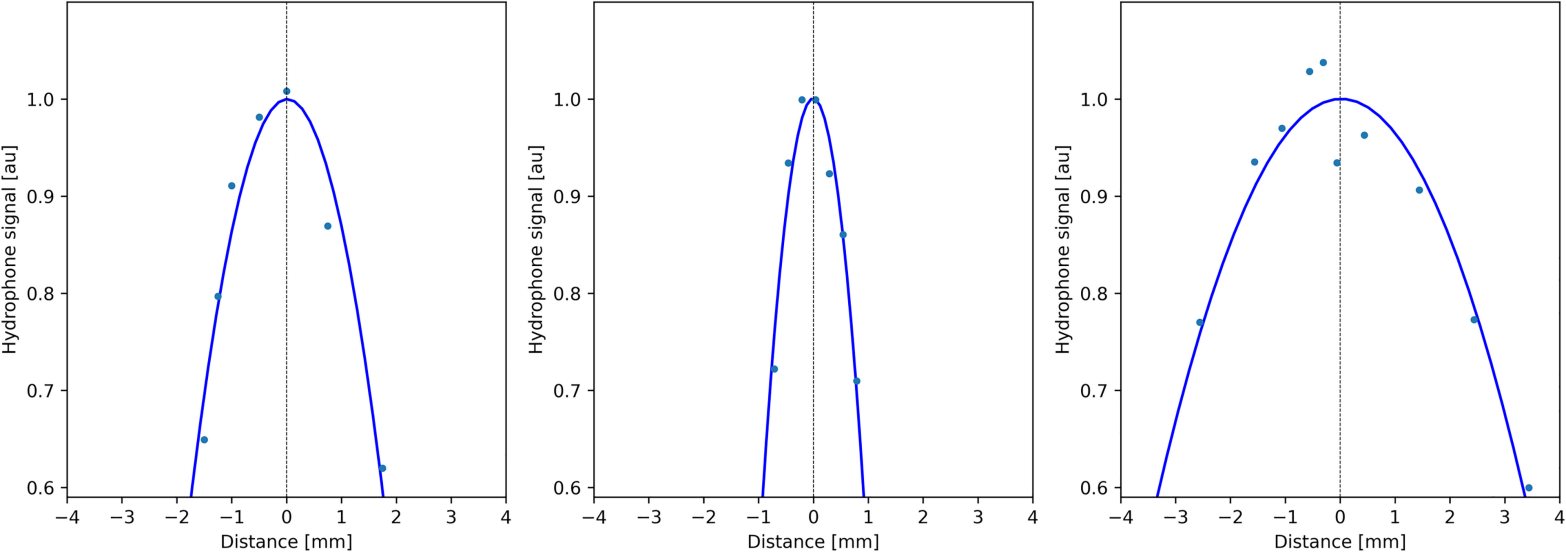
Profiles of acoustic pressure amplitude measured by a thin hydrophone scanning along the symmetry directions of the transducer (*X*, *Y*, and *Z*; see **Figure 1A**), demonstrating the size of the focal spot.

Quantitative analysis of temperature maps confirmed the absence of significant secondary lobes. The focal spot intensity FWHM was found (3.1, 1.6, and 5.7 mm, respectively, for the *X*-, *Y*-, and *Z*-axes) (see **Figure 4**), based on hydrophone measurements. The numerical computing predicted 2.1, 1.1, and 4.0 mm, respectively.

At the end of a 40-s continuous sonication, the temperature elevation of the piezocomposite material measured by the embedded thermocouples at the most critical region of the transducer was 9.6°C at 700 W and 13.5°C at 900 W.

### *In-Vivo* Studies

The animal experiments demonstrated the technical feasibility of the intervention in terms of conforming the body surface, air bubble-free coupling of the device to the skin, space management inside the MR tunnel, targeting of the liver, and deep tissue ablation, as well as cooling water circulation and the absence of interferences between the MR scanner and HIFU transducer up to 900 W.

**Figure 5:**
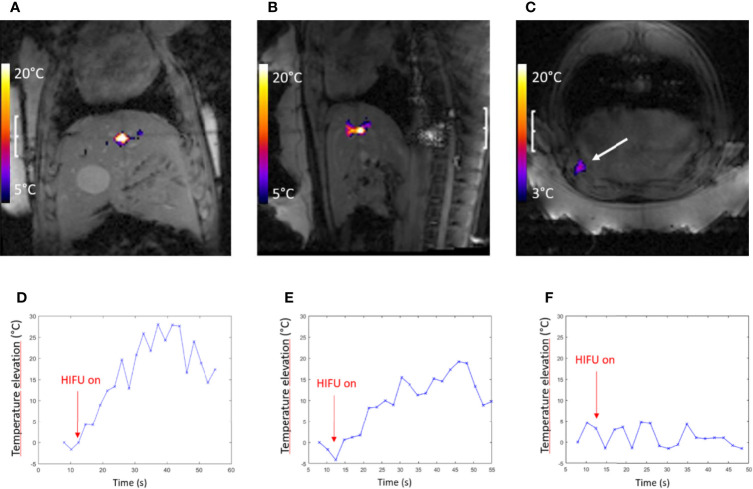
Highlights of pig #1 experiment. PRFS MR temperature map merged with the magnitude image: the FOV is 240 mm^2^, illustrating the temperature elevation at the focal point, coronal **(A)** and sagittal **(B)** plane, the end point of sonication (*t* = 30 s). **(C)** Axial plane, monitoring the most exposed rib (see arrow). A color bar of temperature elevation is embedded. **(D, E)** The highest temperature elevation in coronal and sagittal slice versus time, respectively, spatially averaged in a 2 × 2-pixel ROI. **(F)** Temperature elevation versus time on the exposed rib shown in frame **(C)**.

As illustrated in **Figures 5A, B, D, E**, the maximum relative temperature elevation in a 4-mm-pixel ROI around the warmest pixel in pig #1 and pig #2 was 28.0°C and 25.0°C, meaning 66°C and 63°C absolute temperature, respectively. Rib temperature analysis in the available PRFS slices (**Figures 5C, F**) showed a maximum temperature elevation of 4.8°C in pig #1 and 5.7°C in pig #2, meaning a thermal contrast ratio higher than 4 between the focus and the ribs, without any special means of protection.

The D7 post-intervention MR examination revealed a thermal lesion at the liver parenchyma target in both pigs without secondary lobes or secondary focal areas. Non-perfused volume size was found in pig #1 and pig #2 of 8/11, 6/8, and 7/7 mm along the LR, AP, and HF directions, respectively.

**Figure 6:**
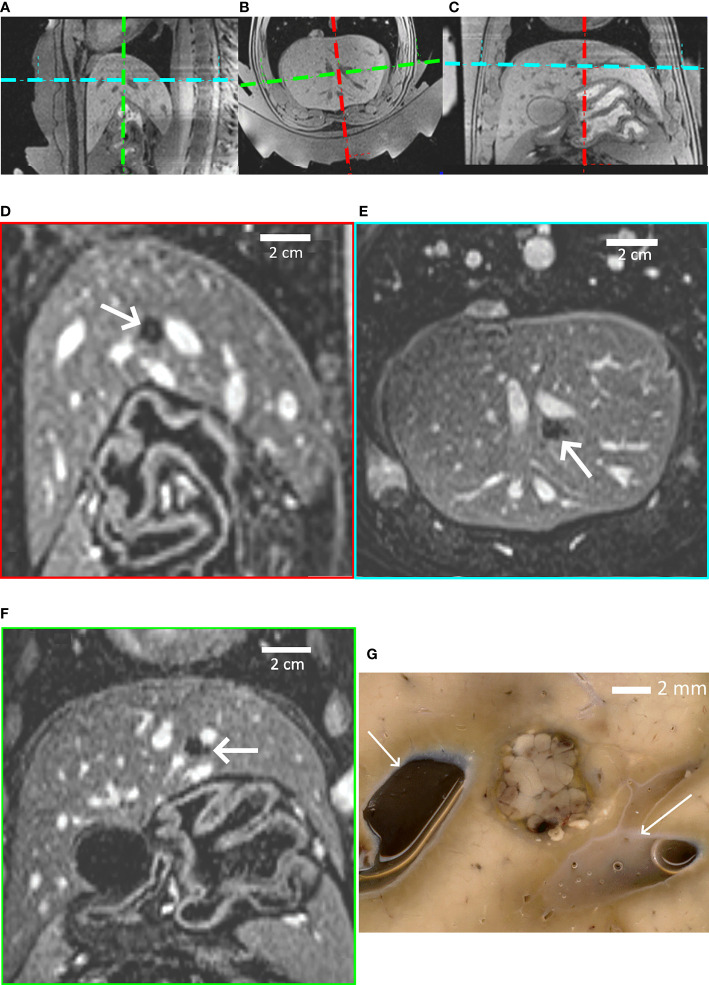
Highlights of pig #1 experiment. **(A–C)** *In-situ* targeting based on high-resolution 3D T1w MR images. Three orthogonal sections are shown. The thermal lesion is prescribed at the crossing point of the transducer’s symmetry axes, visualized with colored dashed lines. **(D–F)** Post-intervention Gd-enhanced MR images illustrating the thermal lesion proximal to subhepatic veins in the sagittal, axial and coronal plane; the FOV is 155 mm. **(G)** Post-mortem gross pathology of the lesion after formalin fixation, confirming the well-defined thermal ablation. White arrows indicate blood vessels. A distance scale is embedded.

**Figure 6** provides further insights regarding pig #1, determining the focal point position from the intersection of the transducer’s symmetry axes (**A–C**), non-perfused volume in liver parenchyma (**D–F**), and gross pathology demonstrating a sharply delineated lesion (**G**).

**Figure 7:**
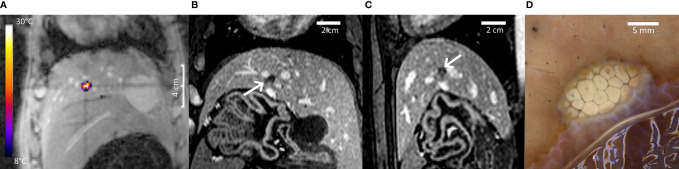
Highlights of pig #2 experiment. **(A)** PRFS MR temperature map merged with the magnitude image at the end point of a 40-s HIFU sonication, coronal plane. A color bar of temperature elevation is provided. **(B, C)** Non-perfused volume on Gd-enhanced MRI, coronal and sagittal. **(D)** Post-mortem gross pathology of the lesion after formalin fixation, confirming the well-defined thermal ablation. A distance scale is embedded in each frame.

**Figure 7** provides further insights regarding pig #2, PRFS MR thermometry in a coronal plane at the end point of sonication (**A**), non-perfused volume in liver parenchyma (**B, C**), and gross pathology (**D**). The intersection of MR thermometry slices defining the prescribed position of the focal point is visible in **Figures 5A, B** and **7A**.

**Figure 8:**
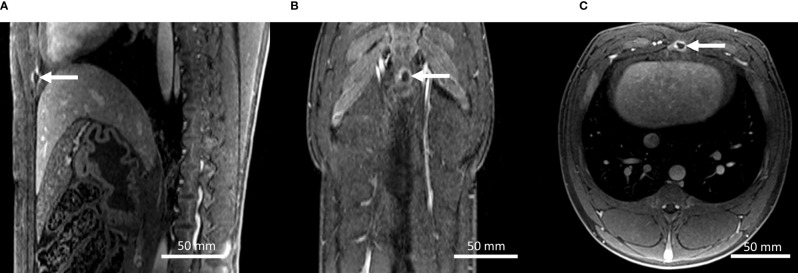
**(A–C)** Gd-enhanced high-resolution T1w MR images in the coronal, axial, and sagittal planes on pig #1, 7 days after the HIFU treatment. A small thermal lesion on the sternum is observed (arrows).

High-resolution contrast-enhanced 3D MRI showed a minor circular lesion on the sternum in both animals, as illustrated in **Figure 8**. Outside the sternum and the focal area, MRI follow-up did not indicate thermal lesions, such as on the rib cage, in the subcapsular, or within the abdominal wall.

Post-mortem visual evaluation of the ribs, peritoneal cavity, and the liver surface based on macroscopic inspection did not reveal any apparent lesions or color change due to accidental HIFU exposure.

## Discussion

Published reports on patients (43–47) have selected a very specific population to target accessible tumors in the liver and pancreas systematically located below the rib line.

HIFU ablation in the liver can lead to adverse events as previously reported by Jung et al. (51, 52) in patients with hepatic cancer, like biliary obstruction, pneumothorax, third-degree burn, rib fracture, or fistula formation between the ablated tumor and an abdominal wall abscess, as well as minor adverse events like adipose edema, skin redness, blisters, pain, or nausea (53, 54).

Previous studies preventing rib burn during liver ablation involved major drawbacks such as longer procedures or restriction on the patient’s eligibility, and some approaches induced a decrease in the energy received at the target compared with the energy delivered. In our study, the transducer design has been optimized to spread the emitted acoustic energy on the skin surface and avoid rib burn without any means of protection. During the procedure, MR thermometry monitored rib hyperthermia, and 7 days after the treatment, Gd-enhanced contrast MR and post-mortem examination confirmed the absence of rib lesions in the two treated animals.

Overall, the design of our HIFU applicator considered various factors and needed to compromise between acoustic requirements, available technologies, and abdominal anatomy. The number of elements was chosen according to the available beam former capacity. A higher number of elements would obviously be beneficial. Therefore, the steering capability was preferentially oriented in the axial plane, where the displacement of the focal point by mechanical means is more difficult, unlike the craniocaudal translation of the applicator according to human anatomy.

The main novelty here is considered the “super-focusing” geometry, yielding a low effective F-number and spreading the near-field acoustic field over a large entry window. Numerical simulations of the acoustic pressure were in millimeter-range agreement with the experimental measurements in terms of shape and size of the focal area.

The ablation in pig #1 and pig #2 confirmed the accuracy of the intervention by the absence of injuries on blood vessels despite their vicinity. The proximal close blood flow did not interfere with the ablation despite the well-described heat-sink effect (55). Our study may open the field of new clinical translation of minimally invasive therapy under magnetic resonance guidance, that is, the transcostal ablation of liver malignancies with a sustainable patient workflow.

However, the current study did not address other obstacles along the beam pathway, such as scars, the sternum, air-filled structures, stomach, or bowel. Scar issues have been addressed by Keserci et al. (54) by adding scar patches on the skin during uterine HIFU ablation leading to minor adverse effects. A bowel-sparing procedure for US-guided abdominal HIFU was reported by Orsi et al. (38). The patient preparation was anticipated 3 days before the treatment, consisting in a liquid diet, enema, fasting 12 h before the procedure, and the use of water balloons to compress the bowels and move the organs at risk outside the beam pathway.

In our study, MRI follow-up and post-mortem inspection revealed a small circular sternal lesion in both pigs. The sternum lesion is suggested to be readily manageable by adding acoustic reflective material on the skin located between the sternum and the beam pathway. Previous studies described the use of acoustic reflective foam pads designed for this purpose (35). The sternal anatomy is significantly simpler and less patient-dependent than the rib cage since a unique median structure has to be shielded. The sternum is found straightforward by palpation and is immobile when the patient is positioned prone. The differences in geometry, blood perfusion, and tissue structure between the ribs and sternum may explain the different sensitivities to the near-field HIFU exposure. This question will be addressed in further *in-vivo* studies.

Furthermore, the sonication duration was limited here by apneas. More elaborated motion compensation strategies are available nowadays; however, we wanted to demonstrate the sharpness of ablation without confounding factors, such as suboptimal motion compensation. In future studies, our novel device will be combined with motion tracking (56) or self-scanning (42) to take advantage of respiratory motion.

While this study confirmed the ability to induce thermal ablation in-depth, a further limitation was not targeting a prescribed location. In other words, we demonstrated the sharpness of the lesions, not their geometric control in space. In future studies, a histological marker (57) may be employed as a ballistic model. The marker should be defined before the HIFU ablation by an interventional radiologist in a plausible region to target liver tumors. The next step would be to target this marker and confirm the accuracy by Gd-enhanced MRI and histologically. Finally, a strategy should be validated for producing larger ablative lesions of several centimeters.

## Conclusion

A novel multisegment super-convergent phased-array transducer has been designed, optimized, and manufactured for deep liver ablation. The validation and calibration tests on the transducer confirmed the performance of the device. Sonications in tissue-mimicking phantoms demonstrated accurate shape of the focal spot, similar to the numerical model. Beam steering experiments confirmed the absence of secondary lobes around the focal point and suitable volumetric focusing capacities.

*In-vivo* experiments in two pigs validated the sharpness of the thermal ablation and the absence of interferences between the devices during concurrent MR acquisition and HIFU emission at high acoustic output. Delivery of a lethal thermal dose at the focus was visualized by MR thermometry during the intervention, and the corresponding lesions were confirmed by Gd-enhanced MRI and post-mortem examination 7 days after the treatment. As a side effect, we only report a minor thermal lesion on the sternum that should be avoided in future studies by adding an external reflective patch in front of the sternum. This study proves that the novel transducer design is capable of transcostal hepatic thermal lesioning in complex situations, such as proximity to blood vessels, without the need for rib protection. This may open the perspective for minimally invasive thermal therapy in liver regions previously limited or restricted by the risk of adverse effects, such as tumors in the right liver lobe and in segments 7 and 8 of the liver dome (58). Additional studies are needed to take advantage of the respiratory motion to create larger lesions (42).

## Data Availability Statement

MR images and MR temperature data presented in this study will be provided on request.

## Ethics Statement

The animal study was reviewed and approved by the Animal Research Committee of Geneva Canton, under the reference GE/95/20.

## Author Contributions

OL, P-AP, ST, and RS were responsible for the intellectual concept of the study. OL, PG, YM’R, AR, ST, and RS performed the literature screening. OL, PG, YM’R, AP, SB, RH, AR, ST, and RS performed the experiments. OL, AP, SB, CC, LC, P-AP and RS interpreted the results. OL, PG, YM’R, AP, SB, CC, RH, LC, P-AP, AR, ST, and RS prepared the manuscript. All authors contributed to the article and approved the submitted version.

## Funding

This study was funded by the Swiss National Foundation of Science (CR320030_185308). This project has received funding from the European Union’s Horizon 2020 research and innovation programme under the Sklodowska-Curie grant agreement No 813766. Open access funding provided by University Of Geneva.

## Conflict of Interest

The authors declare that the research was conducted in the absence of any commercial or financial relationships that could be construed as a potential conflict of interest.

## Publisher’s Note

All claims expressed in this article are solely those of the authors and do not necessarily represent those of their affiliated organizations, or those of the publisher, the editors and the reviewers. Any product that may be evaluated in this article, or claim that may be made by its manufacturer, is not guaranteed or endorsed by the publisher.
